# Supplementary material for: Adult microglial TGFβ1 is required for microglia homeostasis via an autocrine mechanism to maintain cognitive function in mice
Source: Nat Commun. 2024 Jun 21;15:5306. doi: 10.1038/s41467-024-49596-0 (PMC11192737; doi:10.1038/s41467-024-49596-0)
Supplement: Supplementary file 1 — Supplementary Information [file 41467_2024_49596_MOESM1_ESM.pdf]

Adult Microglial TGFβ1 is critical for microglia homeostasis via an autocrine mechanism to maintain cognitive function in mice

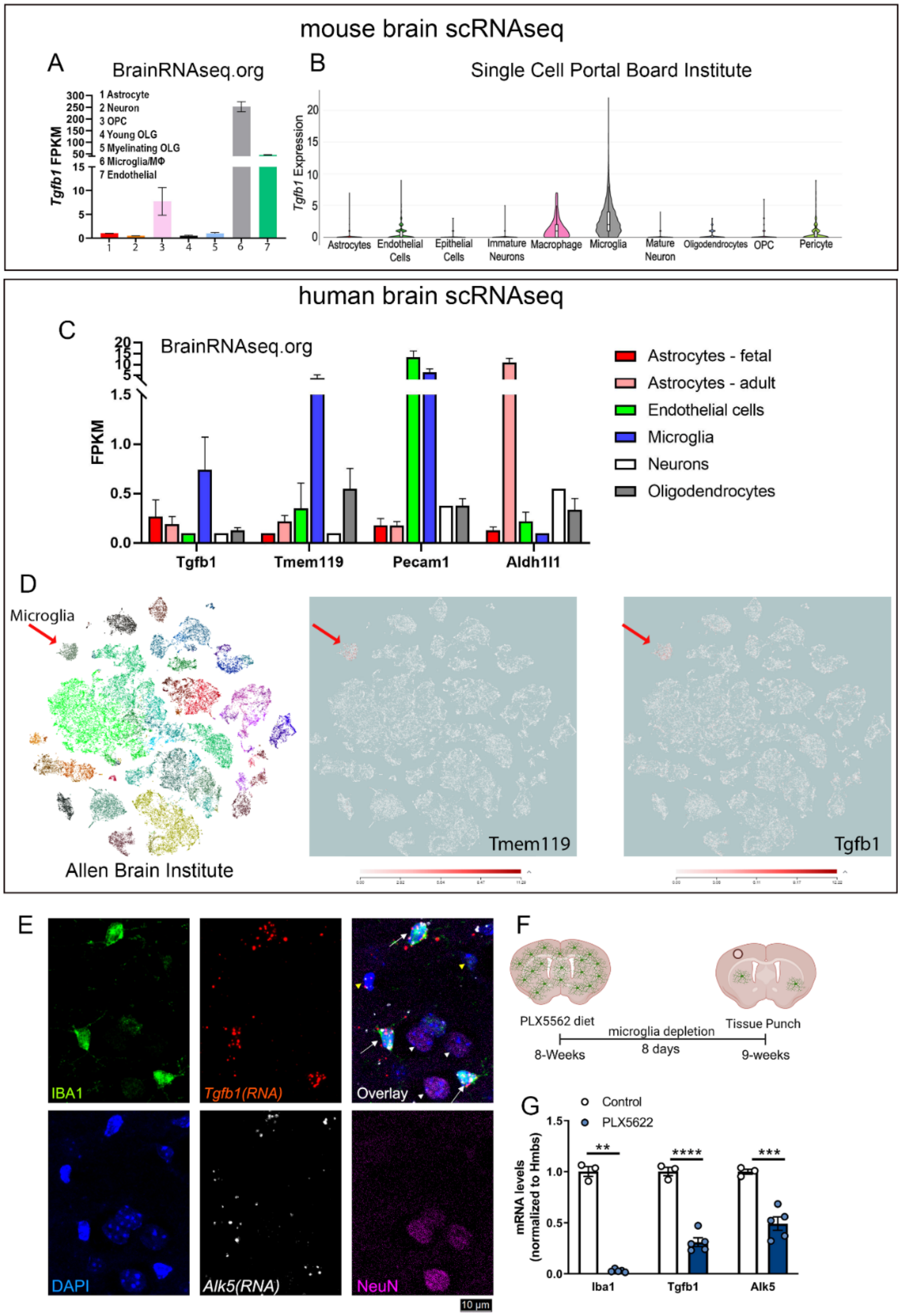

Supplementary Figure 1. ***Tgfb1*** **gene expression is enriched in microglia in adult mice and human brains.** (A, B) Publicly available single-cell RNA-sequencing datasets support microglial enrichment for *Tgfb1* expression from Zhang et. al 2014 (A) showing fragments per kilobase of transcript per million reads mapped (FPKM) and a second independent Single-cell portal data set[1] (B) showing similar microglial enrichment of the *Tgfb1* expression among different cell types in mouse brain. (C, D) Similarly, publicly available single-cell

RNAseq datasets at Brainrnaseq.org (C) and Allen Brain Institute (D) also show similar enrichment of microglial *Tgfb1* gene expression in the human brain dataset. (E) Representative images using combined RNA-scope/Immunohistochemistry visualizing RNA-scope in situ signal for *Tgfb1* mRNA and *Tgfbr1* (*Alk5)* mRNA combined with IHC for IBA1 and NEUN showing colocalization of *Tgfb1* and *Alk5* mRNA with IBA1+ microglia (white arrow) but not in NEUN+ neurons (white arrowhead). Note some *Tgfb1* mRNA signals in IBA1- cells (yellow arrowhead). (F) Experimental timeline for PLX5622 ablation of microglia, (G) microglia ablation leads to a significant decrease of *Iba1*, *Tgfb1*, and *Alk5* mRNA levels in total brain tissue (normalized to *Hmbs1*). Data from cortical tissue was shown and striatal tissue showed similar results. Mean$\pm$SE, ** = $p<0.001$, *** = $p<0.0005$, **** = $p<0.0001$, unpaired Student's t-test, 2-sided. (each data point represents an individual animal). Supplementary Figure 1F was created with Biorender.com released under a Creative Commons Attribution-NonCommercial-NoDerivs 4.0 International license.

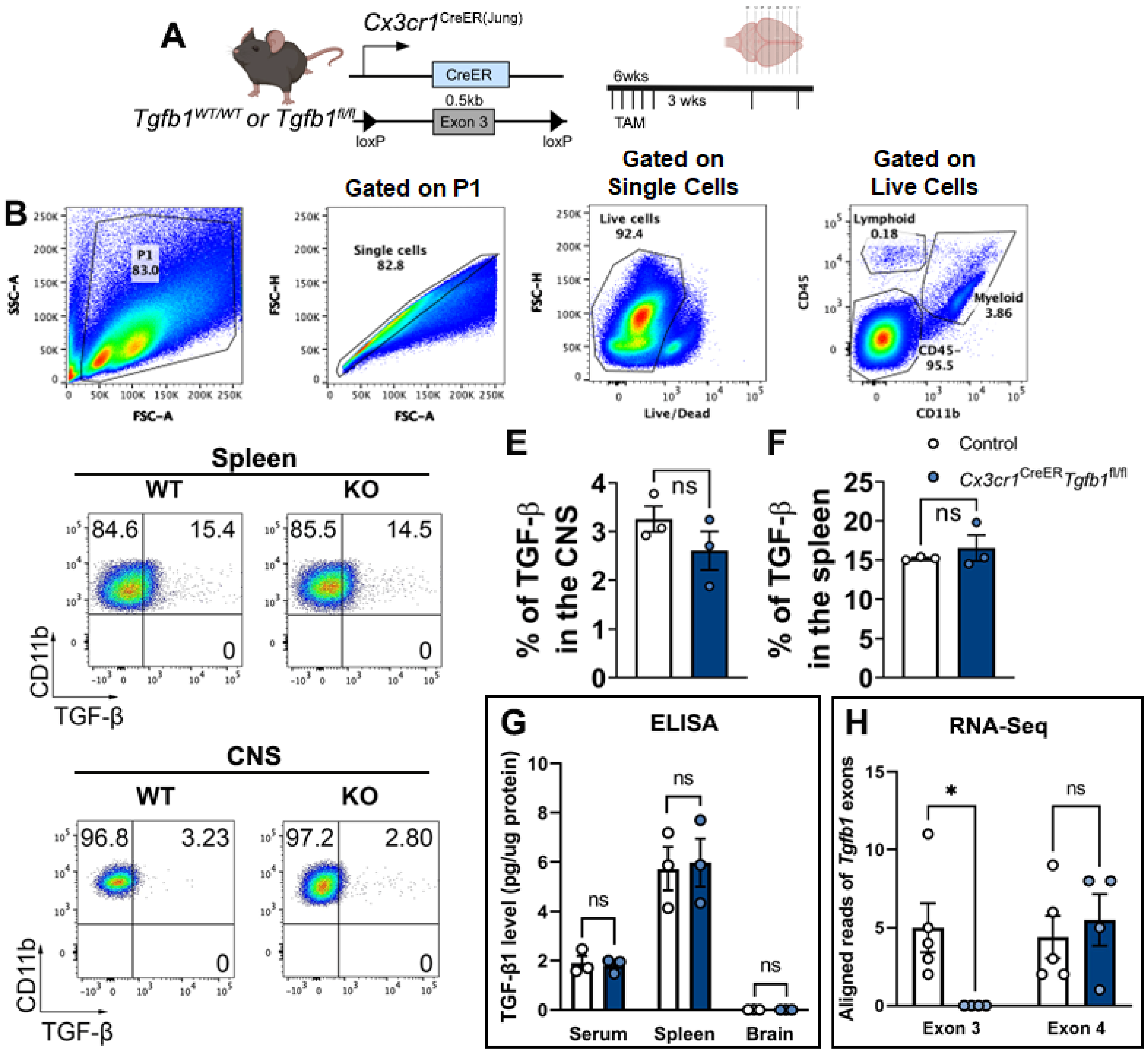

Supplementary Figure 2. FACS or ELISA detection of TGF-β1 protein in the brain, serum, and spleen in WT or MG-*Tgfb1* iKO mice. (A) Timeline for using *Cx3cr1*^{CreER} driver to target myeloid cell inducible *Tgfb1* loss (samples were harvested at 3 weeks post TAM). (B) Gating strategy for identifying the myeloid cells in single-cell suspension. Cells were first gated according to FSC-SSC, then restricted to single and live cells. Myeloid cells were identified as CD45+ CD11b+. (C, D) FACS analyses of TGF- β expression by myeloid cells in the spleen (top histograms) and in the CNS (lower histograms, whole brain except cerebellum). (E, F) Compilation of TGF-β expression by flow cytometry on myeloid cells from the spleen and CNS of WT and KO mice. (G) ELISA quantification (pg/µg total protein) from serum and tissue from the spleen and whole brain (n = 3 mice per group) showing no difference in TGF-β protein levels in serum or spleen of *Cx3cr1*^{CreER}*Tgfb1* iKO or control mice and that the brain TGF-β ligand levels are below the detection limit of the kit. (H) Aligned reads from bulk RNA-sequencing data from whole brain microglia showing decreased exon 3 (Floxed exon) reads in the *Cx3cr1*^{CreER}*Tgfb1* iKO but not exon 4 (non-floxed exon). Mean$\pm$SE, unpaired Student's t-test, 2-sided, (each data point represents a single animal). Supplementary Figure 2A was created with Biorender.com released under a Creative Commons Attribution-NonCommercial-NoDerivs 4.0 International license.

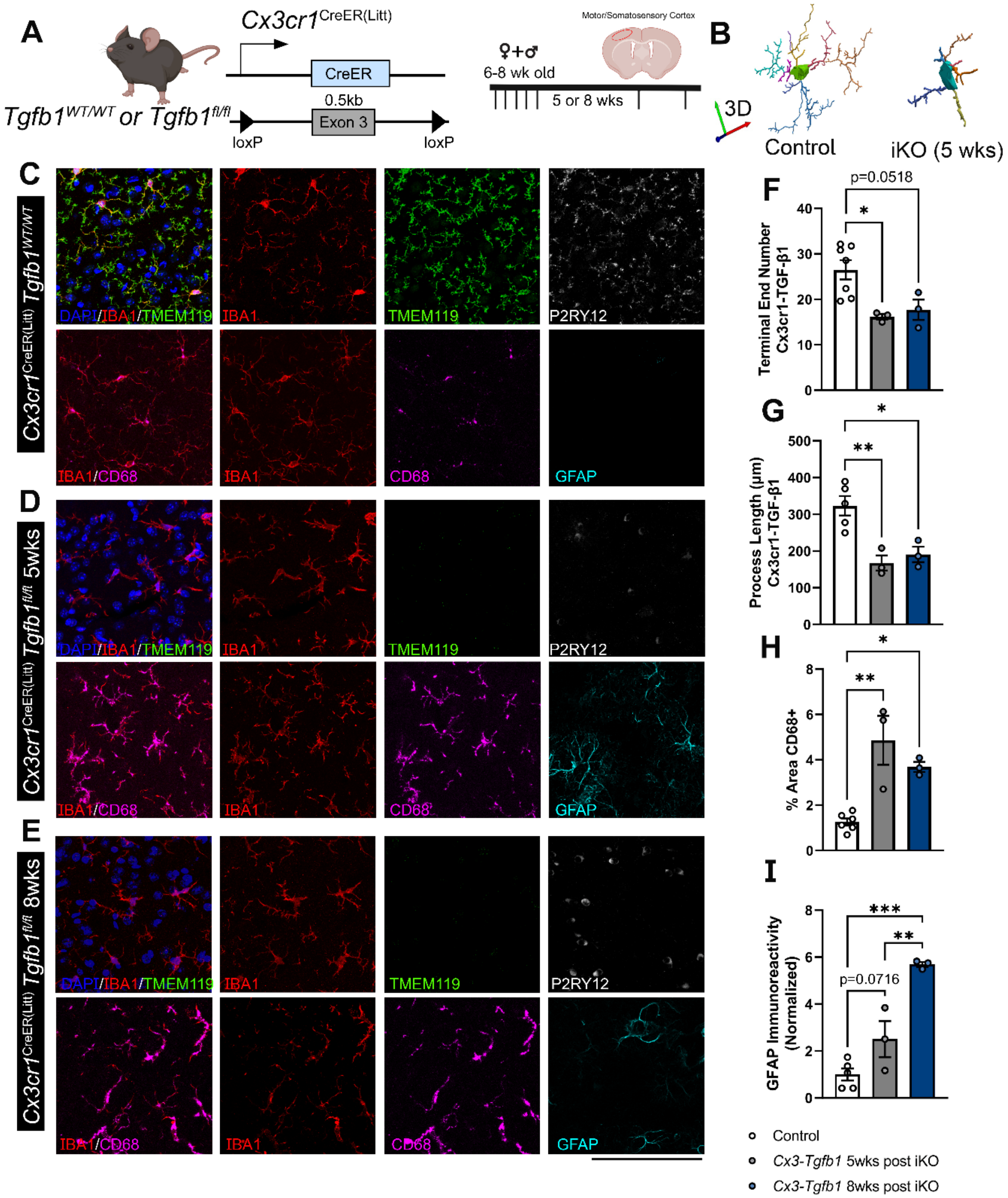

Supplemental Figure 3. Microglia-specific *Tgfb1* gene deletion results in loss of homeostasis of microglia and increased reactive astrocytes in the cortex of the adult mouse brain. (A) A mouse model for targeting microglial *Tgfb1* and experimental timeline. (B) 3D reconstruction of control and iKO microglia. Representative immunohistochemistry images of IBA1, TMEM119, P2RY12, CD68, and GFAP in the cortex of (C) Control animals, (D) *Cx3cr1*$^{CreER(Litt)}$*Tgfb1*$^{fl/fl}$ knockouts 5 weeks after tamoxifen administration, and (E) *Cx3cr1*$^{CreER(Litt)}$*Tgfb1*$^{fl/fl}$ knockouts 8 weeks after tamoxifen administration. Quantification of (F) microglial process terminal end numbers, (G) total microglial process length, (H) CD68 immunoreactivity, and (I) GFAP immunoreactivity. Mean$\pm$SE, * = $p<0.05$, ** = $p<0.01$, *** = $p<0.001$. One-way ANOVA, 2-sided. (> 40 microglia were quantified for each animal and the average from one mouse was plotted as a single data point in the figure panel and treated as n=1 for statistical analysis). Both females and males are included and we did not observe a sex difference in our analysis. Scale bar = 100µm. Supplementary Figure 3A was created with Biorender.com released under a Creative Commons Attribution-NonCommercial-NoDerivs 4.0 International license.

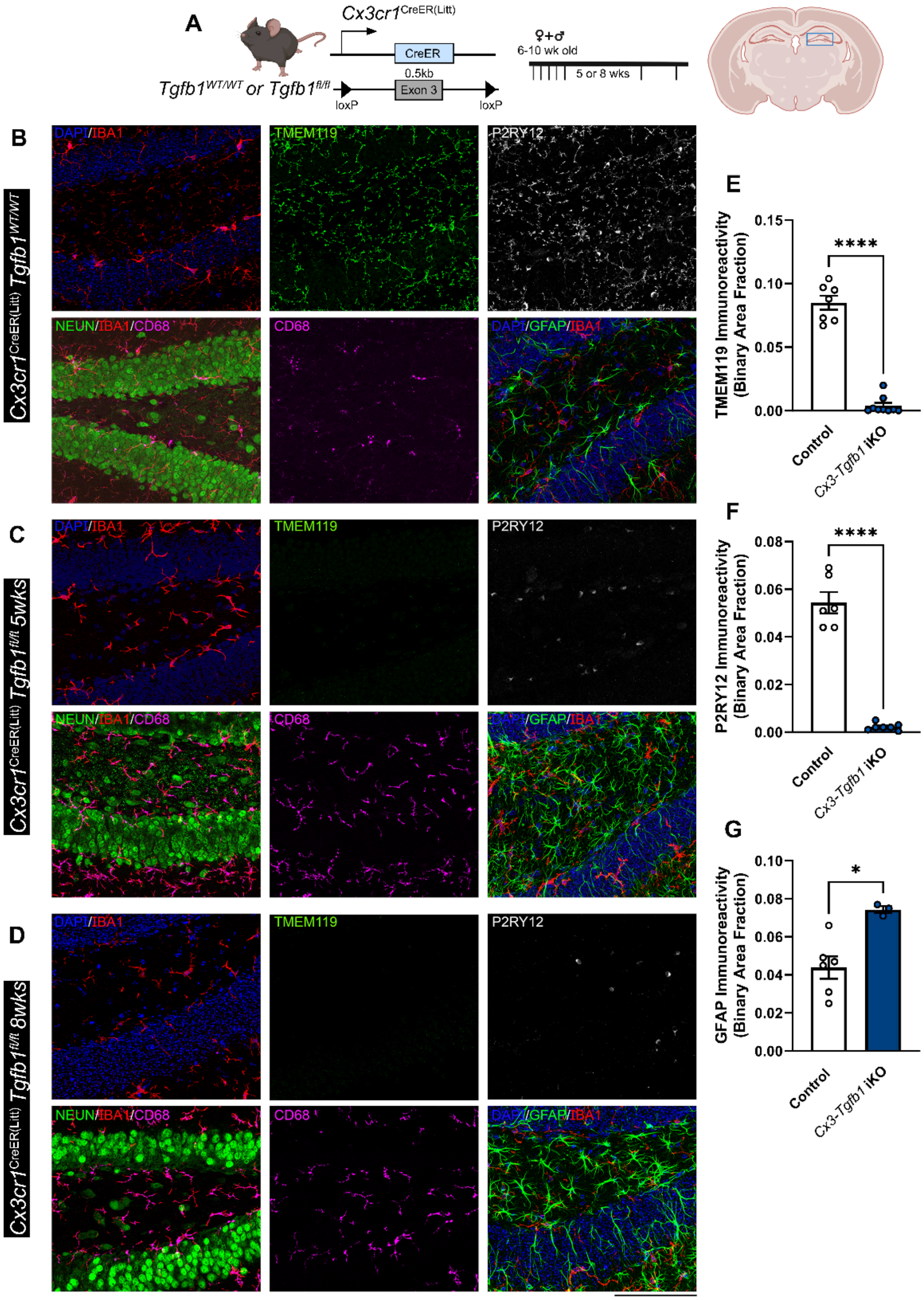

Supplemental Figure 4. Microglia-specific *Tgfb1* gene deletion results in loss of homeostasis of microglia and an increase in reactive astrocytes in the hippocampus of the adult mouse brain. (A) Mouse model for targeting microglial *Tgfb1* and experimental timeline. (B) Representative immunohistochemistry images of IBA1, TMEM119, P2RY12, CD68, NeuN, and GFAP in the hippocampus (dentate gyrus region shown) of control animals, (C) *Cx3cr1*$^{\mathrm{CreER(Litt)}}$*Tgfb1*$^{\mathrm{fl/fl}}$ knockouts 5 weeks after tamoxifen administration, and (D) *Cx3cr1*$^{\mathrm{CreER(Litt)}}$*Tgfb1*$^{\mathrm{fl/fl}}$ knockouts 8 weeks after tamoxifen administration. Quantification of (E) TMEM119 immunoreactivity, (F) P2RY12 immunoreactivity, and (G) GFAP immunoreactivity. Representative results from n=3-6 mice/group, iKO 5 wk and 8 wks groups have no difference and are combined as the iKO group. Mean±SE, * = $p<0.05$, **** = $p<0.0001$, unpaired Student's t-test, 2-sided. Scale bar = 100μm. Supplementary Figure 4A was created with Biorender.com released under a Creative Commons Attribution-NonCommercial-NoDerivs 4.0 International license.

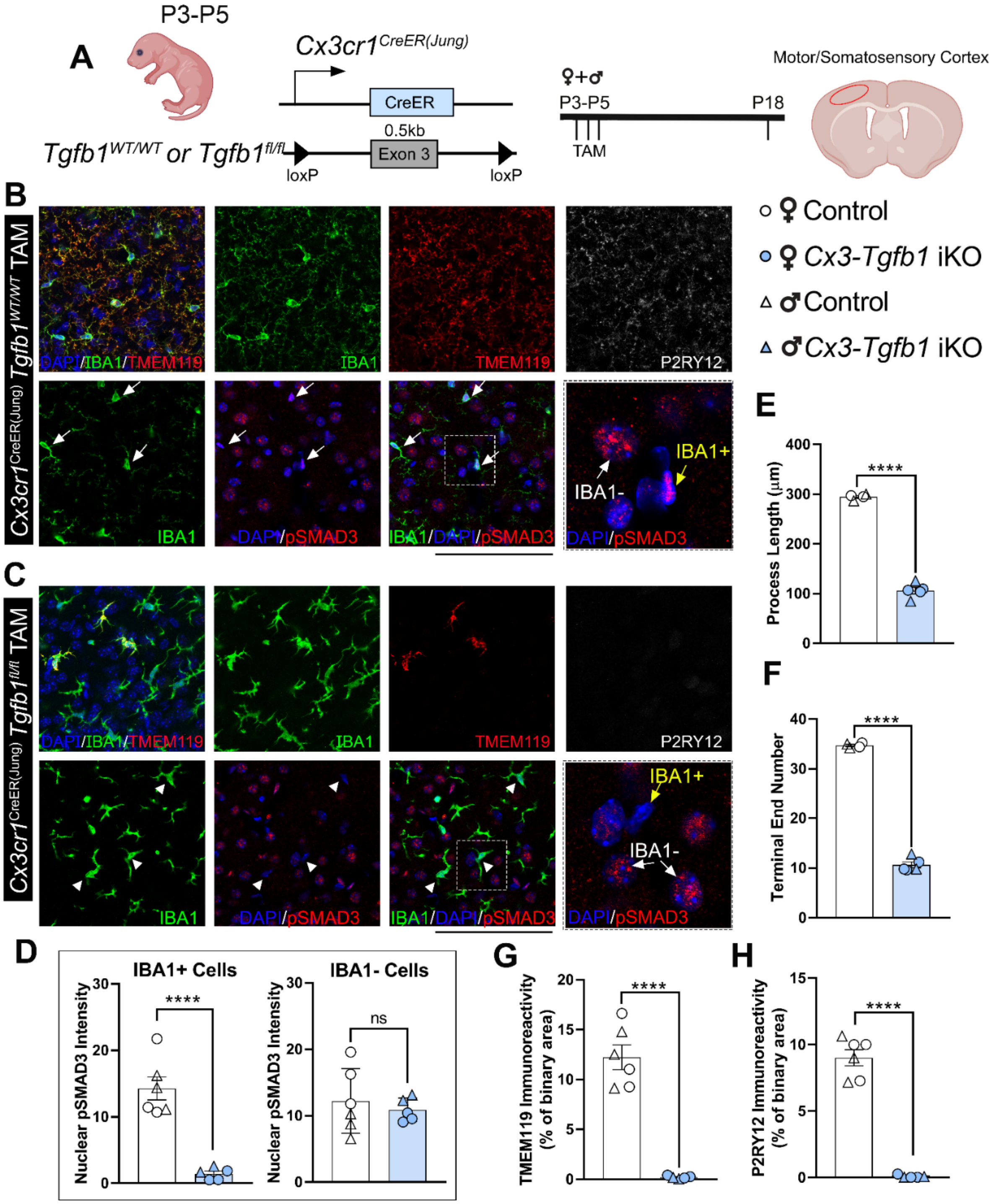

Supplementary Figure 5. Microglia-specific *Tgfb1* gene deletion results in loss of homeostasis of microglia in the neonatal mouse brain (somatosensory cortical region shown as representative). (A) A mouse model for targeting microglial *Tgfb1* and experimental timeline. Representative immunohistochemistry images of IBA1, TMEM119, P2RY12, pSMAD3 in the cortex of (B) Control animals, (C) *Cx3cr1*$^{\mathrm{CreER(Litt)}}$*Tgfb1*$^{\mathrm{fl/fl}}$ knockouts ~3 weeks after tamoxifen administration. Quantification of (D) nuclear pSMAD3 immunoreactivity in IBA1+ and IBA1- cells. (E) total microglial process length (F) microglial process terminal end numbers, (G) TMEM119 immunoreactivity, and (H) P2RY12 immunoreactivity in the control and MG-*Tgfb1* iKO neonatal mice Mean$\pm$SE, * = p<0.05, ** = p<0.01, *** = p<0.001. One-way ANOVA, 2-sided. (> 40 microglia were quantified for each animal and the average from one mouse was plotted as a single data point in the figure panel and treated as n=1 for statistical analysis). Both females and males are included, and we did not observe a sex difference in our analysis. Scale bar = 100µm. Supplementary Figure 5A was created with Biorender.com released under a Creative Commons Attribution-NonCommercial-NoDerivs 4.0 International license.

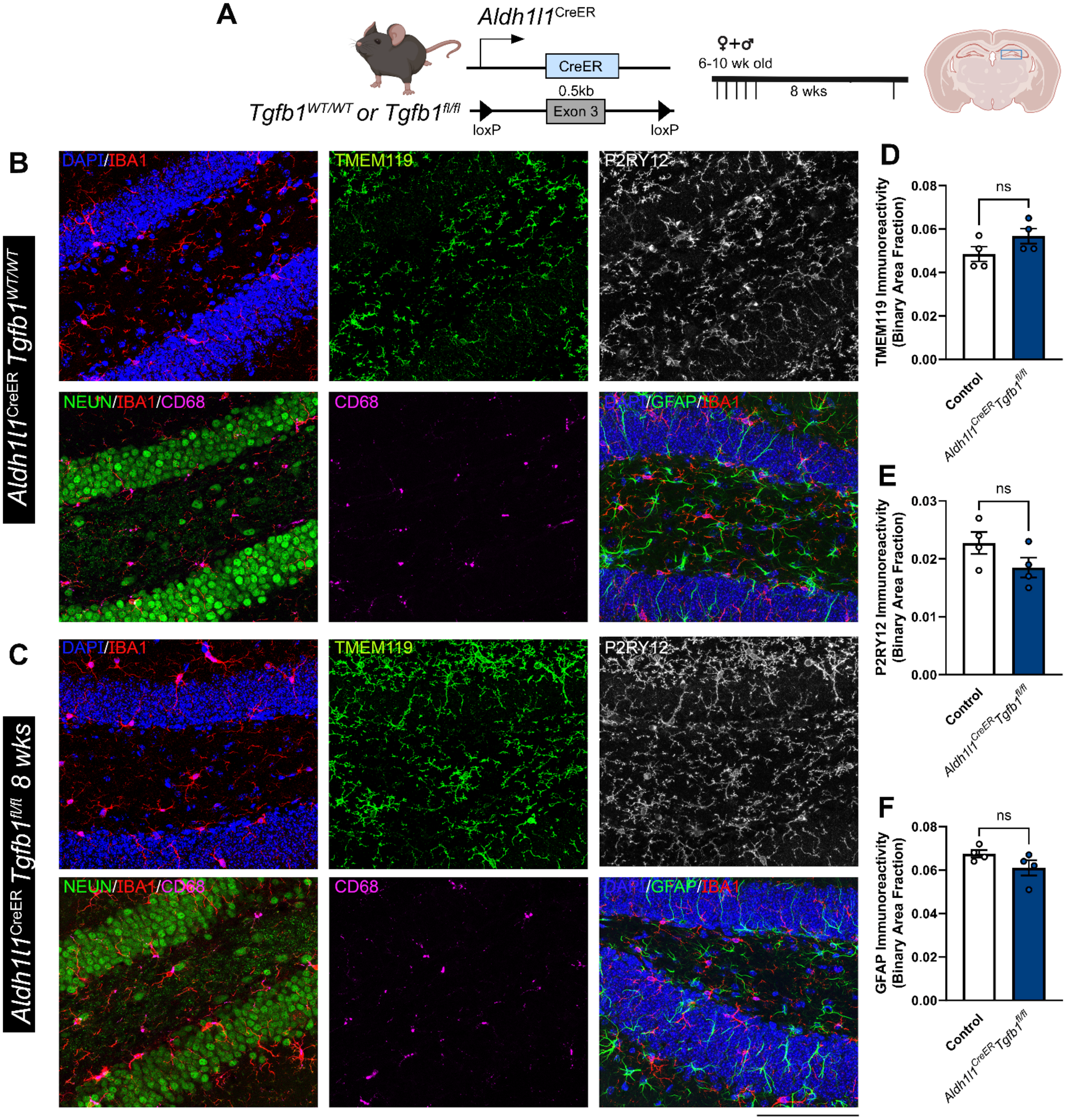

Supplemental Figure 6. Astrocyte-specific *Tgfb1* gene deletion in the *Aldh1l1*^{CreER} driver does not affect the homeostasis of microglia or GFAP expression in astrocytes in the adult mouse brain (hippocampus, dentate gyrus). (A) Astrocyte iKO mouse model and experimental timeline. (B,C) Representative immunohistochemistry images of hippocampus from TAM treated (8 weeks post) control (B) *Aldh1l1*^{CreER}*Tgfb1*$^{wt/wt}$ and (C) iKO *Aldh1l1*^{CreER} *Tgfb1*$^{fl/fl}$ tissue showing IBA1, TMEM119, P2RY12, NEUN, CD68, and GFAP immunostaining. Quantification of (D) TMEM119 immunoreactivity, (E) P2RY12 immunoreactivity, and (F) GFAP immunoreactivity. Representative results from n=3-5 mice/group, Mean±SE, ns= not significant, unpaired Student's t-test, 2-sided, Scale bar = 100μm. Supplementary Figure 6A was created with Biorender.com released under a Creative Commons Attribution-NonCommercial-NoDerivs 4.0 International license.

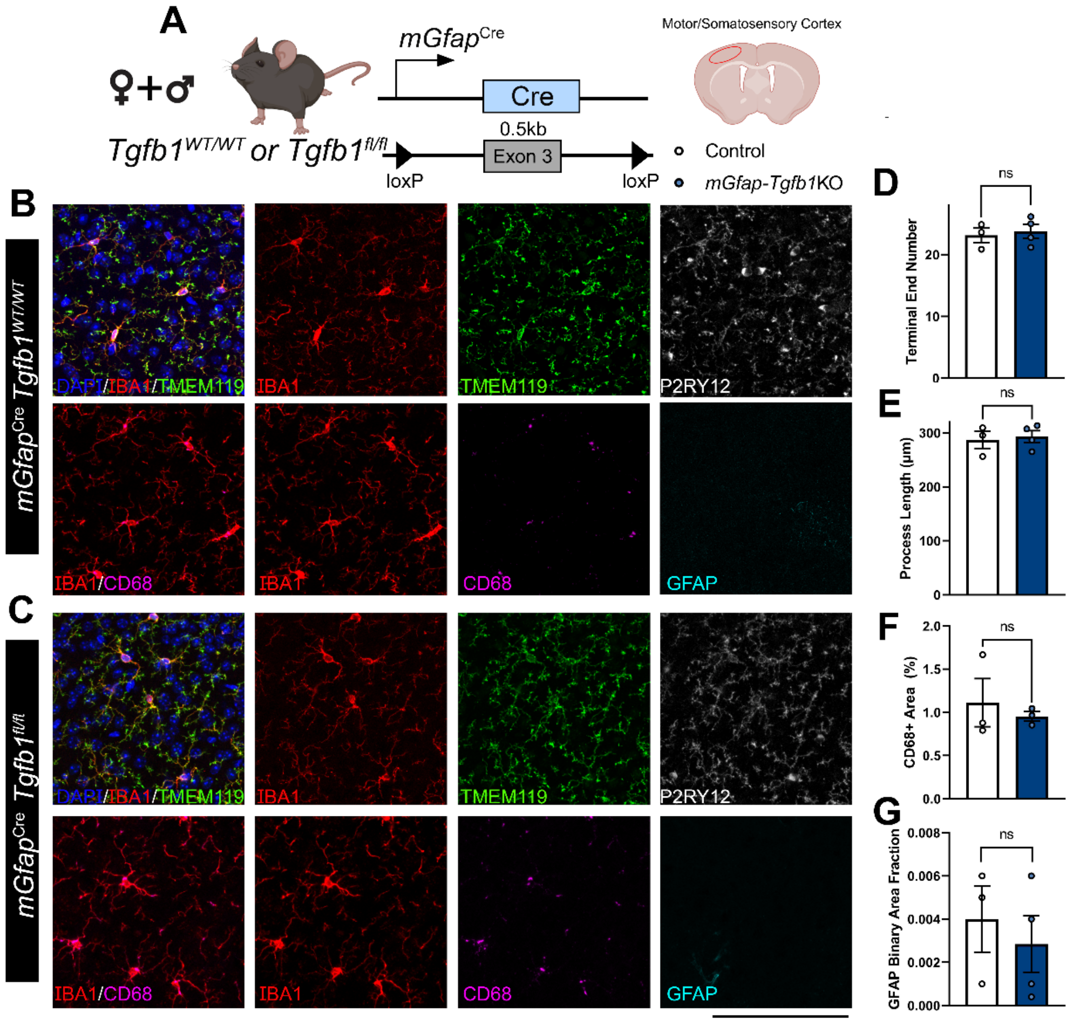

Supplementary Figure 7. Astrocyte-specific *Tgfb1* gene deletion in the perinatal conditional mGfap^{Cre} driver line does not affect the homeostasis of microglia or GFAP expression in astrocytes in the adult (8-week-old) mouse brain (cortex). (A) Astrocyte constitutive KO mouse model used and experimental timeline. (B,C) Representative images of control *mGfap*^{Cre}*Tgfb1*$^{wt/wt}$ (B) and cKO *mGfap*^{CreER} *Tgfb1*$^{fl/fl}$ tissue showing IBA1, TMEM119, P2RY12, CD68, and GFAP immunostaining. Quantification of microglia ramification via (D) terminal end number, (E) process length, and (F) CD68+ immunoreactive % area. (G) Quantification of astrocyte reactivity using GFAP+ immunoreactive area fraction. (control n=3: cKO n=4) ns=not significant. unpaired Student's t-test, 2-sided. Mean+SE, Scale bar = 100µm. Supplementary Figure 7A was created with Biorender.com released under a Creative Commons Attribution-NonCommercial-NoDerivs 4.0 International license.

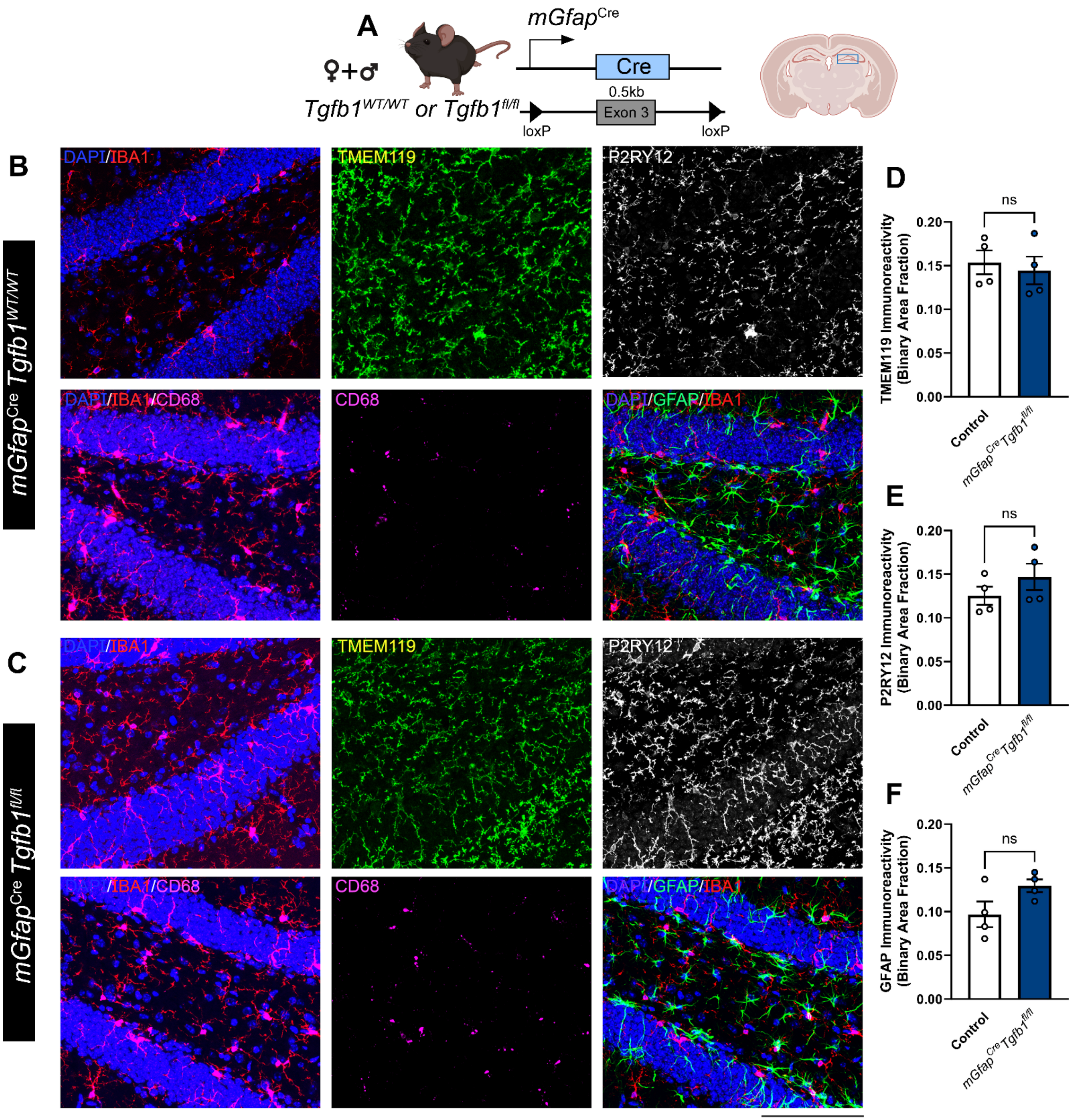

Supplemental Figure 8. Astrocyte-specific *Tgfb1* gene deletion in the perinatal constitutive *mGfap*^{Cre} driver line does not affect the homeostasis of microglia or GFAP expression in astrocytes in the adult (8-week-old) mouse brain (hippocampus, dentate gyrus). (A) Astrocyte constitutive KO mouse model used and experimental timeline. (B, C) Representative images of control *mGfap*^{Cre}*Tgfb1*$^{wt/wt}$ (B) and cKO *mGfap*^{CreER} *Tgfb1*$^{fl/fl}$ tissue showing IBA1, TMEM119, P2RY12, CD68, and GFAP immunostaining. Quantification of (D) TMEM119 immunoreactivity, (E) P2RY12 immunoreactivity, and (F) GFAP immunoreactivity. Mean+SE. ns= not significant. Unpaired Student's t-test, 2-sided. Representative results from n=3-5 mice/group. Scale bar = 100µm. Supplementary Figure 8A was created with Biorender.com released under a Creative Commons Attribution-NonCommercial-NoDerivs 4.0 International license.

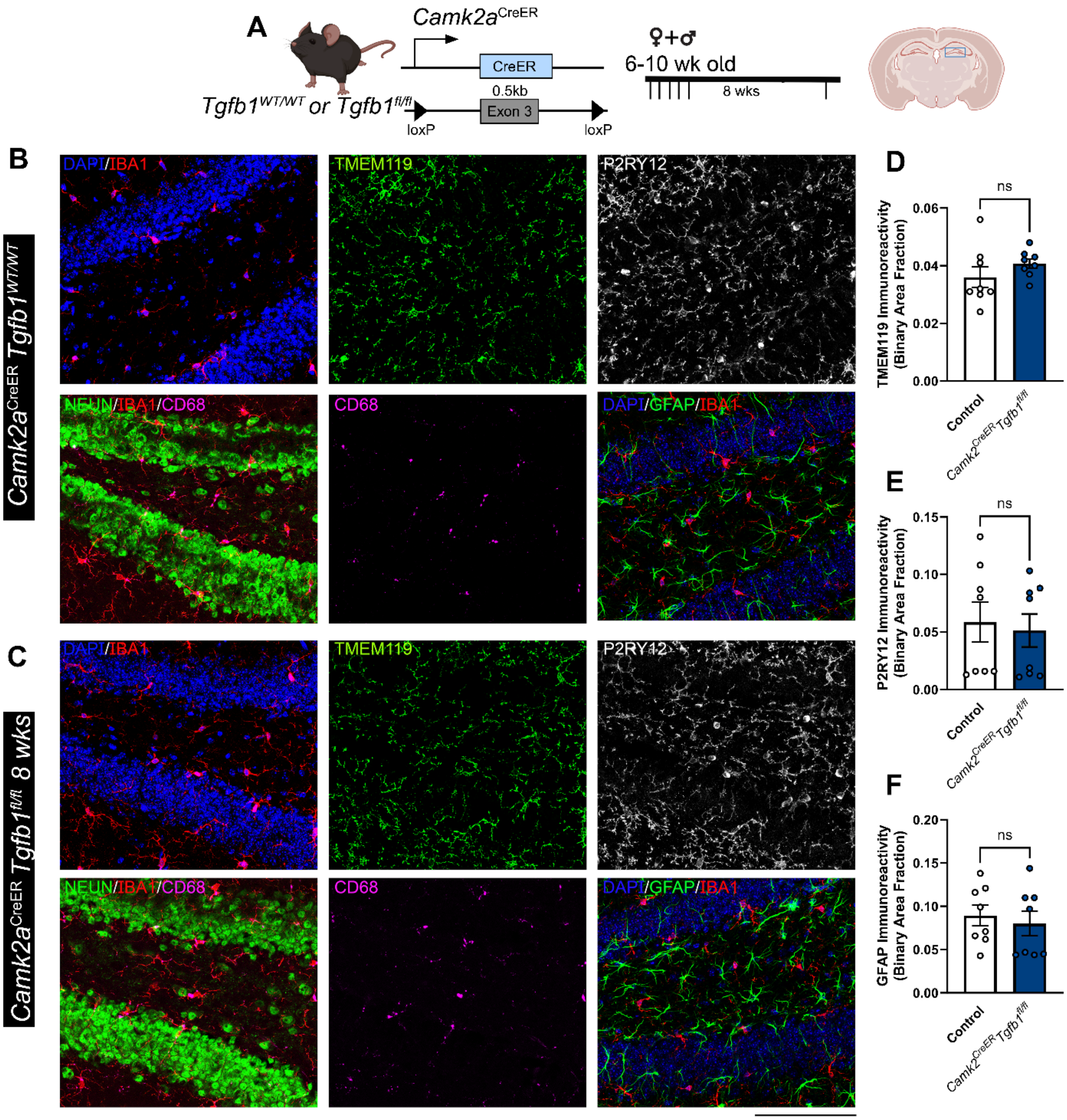

Supplementary Figure 9. Forebrain neuronal specific *Tgfb1* gene deletion in the *Camk2a*^{CreER} driver does not affect the homeostasis of microglia or GFAP expression in astrocytes in the adult mouse brain (hippocampus, dentate gyrus). (A) Neuronal iKO mouse model used and experimental timeline. (B, C) Representative images of control *Camk2a*^{Cre}*Tgfb1*$^{wt/wt}$ (B) and iKO *Camk2*^{CreER} *Tgfb1*$^{fl/fl}$ tissue showing IBA1, TMEM119, P2RY12, NEUN, CD68, and GFAP immunostaining. Quantification of (D) TMEM119 immunoreactivity, (E) P2RY12 immunoreactivity, and (F) GFAP immunoreactivity. Mean+SE. ns= not significant. Unpaired Student's t-test, 2-sided. Scale bar = 100µm. Supplementary Figure 9A was created with Biorender.com released under a Creative Commons Attribution-NonCommercial-NoDerivs 4.0 International license.

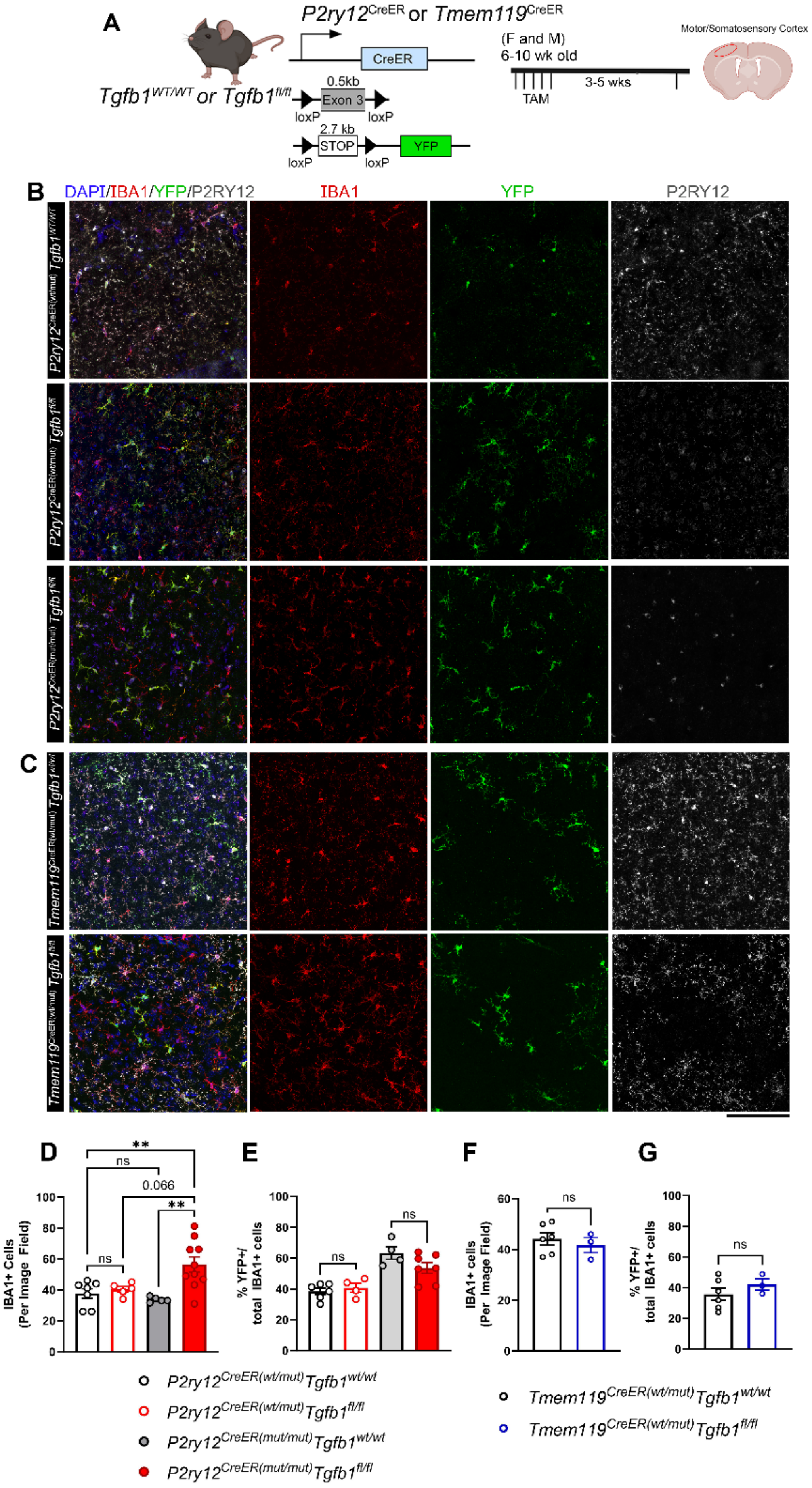
A
P2ry12CreER or Tmem119CreER
CreER
(F and M)
6-10 wk old
Motor/Somatosensory Cortex
Tgfb1WT/WT or Tgfb1fl/fl
0.5kb
Exon 3
loxP
loxP
3-5 wks
TAM
2.7 kb
STOP
YFP
loxP
loxP
B
DAPI/IBA1/YFP/P2RY12
IBA1
YFP
P2RY12
C
D
E
F
G
IBA1+ Cells (Per Image Field)
% YFP+/ total IBA1+ cells
IBA1+ cells (Per Image Field)
% YFP+/ total IBA1+ cells
ns
0.066
**
P2ry12CreER(wt/mut)Tgfb1wt/wt
P2ry12CreER(wt/mut)Tgfb1fl/fl
P2ry12CreER(mut/mut)Tgfb1wt/wt
P2ry12CreER(mut/mut)Tgfb1fl/fl
Tmem119CreER(wt/mut)Tgfb1wt/wt
Tmem119CreER(wt/mut)Tgfb1fl/fl

Supplemental Figure 10: YFP reporter tracking in the microglia-specific iKO lines supports microglia converting to dyshomeostasis rather than the infiltration of peripheral immune cells to replace dying dyshomeostatic microglia. (A) *P2ry12*^{CreER} or *Tmem119*^{CreER} mouse driver to induce *Tgfb1* KO in P2RY12+ or TMEM119+ microglia and experimental timeline. All lines contain one copy of the R26-YFP reporter allele. (B-C) Representative images showing immunohistochemistry staining for DAPI, IBA1, YFP, and P2RY12 expression in (B) *P2ry12*$^{CreER(wt/mut)}$*Tgfb1*$^{WT/WT}$, *P2ry12*$^{CreER(wt/mut)}$*Tgfb1*$^{fl/fl}$, and *P2ry12*$^{CreER(mut/mut)}$*Tgfb1*$^{fl/fl}$ and (C) *Tmem119*$^{CreER(wt/mut)}$*Tgfb1*$^{WT/WT}$ and *Tmem119*$^{CreER(wt/mut)}$*Tgfb1*$^{fl/fl}$. (D) Quantification of total IBA1+ cells and (E) % YFP+ cells among total IBA1+ cells in the *P2ry12*^{CreER} lines and (F) Quantification of total IBA1+ cells and (G) % YFP+ cells among total IBA1+ cells in the *Tmem119*^{CreER} lines. Mean$\pm$SE. **, p<0.01 and ns= not significant. For comparing two groups an unpaired Student's t-test (2-sided) was used and for comparing four groups, a one-way ANOVA (2-sided) analysis was used. Scale bar = 100µm. Supplementary Figure 10A was created with Biorender.com released under a Creative Commons Attribution-NonCommercial-NoDerivs 4.0 International license.

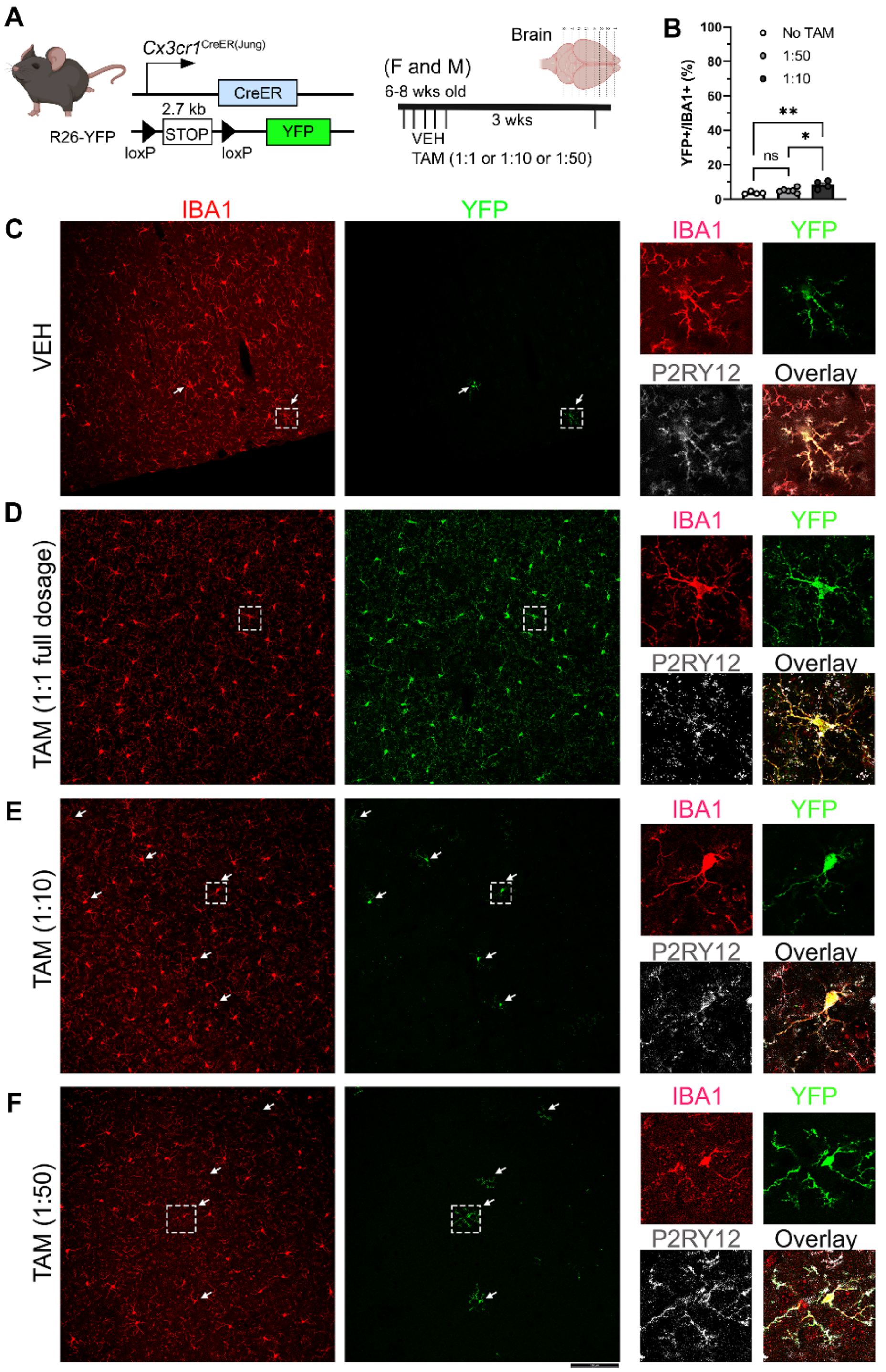

Supplemental Figure 11. Titration of Tamoxifen dosage to enable sparse recombination of a floxed allele in single sparse microglia in adult mouse brain. (A) The reporter mouse model used to test recombination efficiency in the floxed reporter allele. (B) Quantification of YFP+ cells present in the two different doses of TAM administration compared to a vehicle control animal. Note, we have previously reported ~90% YFP+/IBA1+ cells in this mouse line using full dosage TAM. (C-F) Representative IHC images showing IBA1, YFP, and P2RY12 immunostaining from (C) vehicle-treated, (D) full TAM (180mg/kg) dosage, (E) 1:7-1:10 TAM dosage, and (F) 1:50 TAM dosage treated brain tissue. We observed similar recombination efficiency which led to sparse labeling in individual microglia in 1:7-1:10 TAM dosage in our study. P2RY12 expression indicates that parenchyma microglia are recombined. All brain regions are used in the analysis due to the sparse labeling. Mean+SE. One-way ANOVA (2-sided) was used to analyze the results. * $p<0.05$, ** $p<0.01$. Representative results from n=3-5 mice/group. Scale Bar = 100μm. Supplementary Figure 11A was created with Biorender.com released under a Creative Commons Attribution-NonCommercial-NoDerivs 4.0 International license.

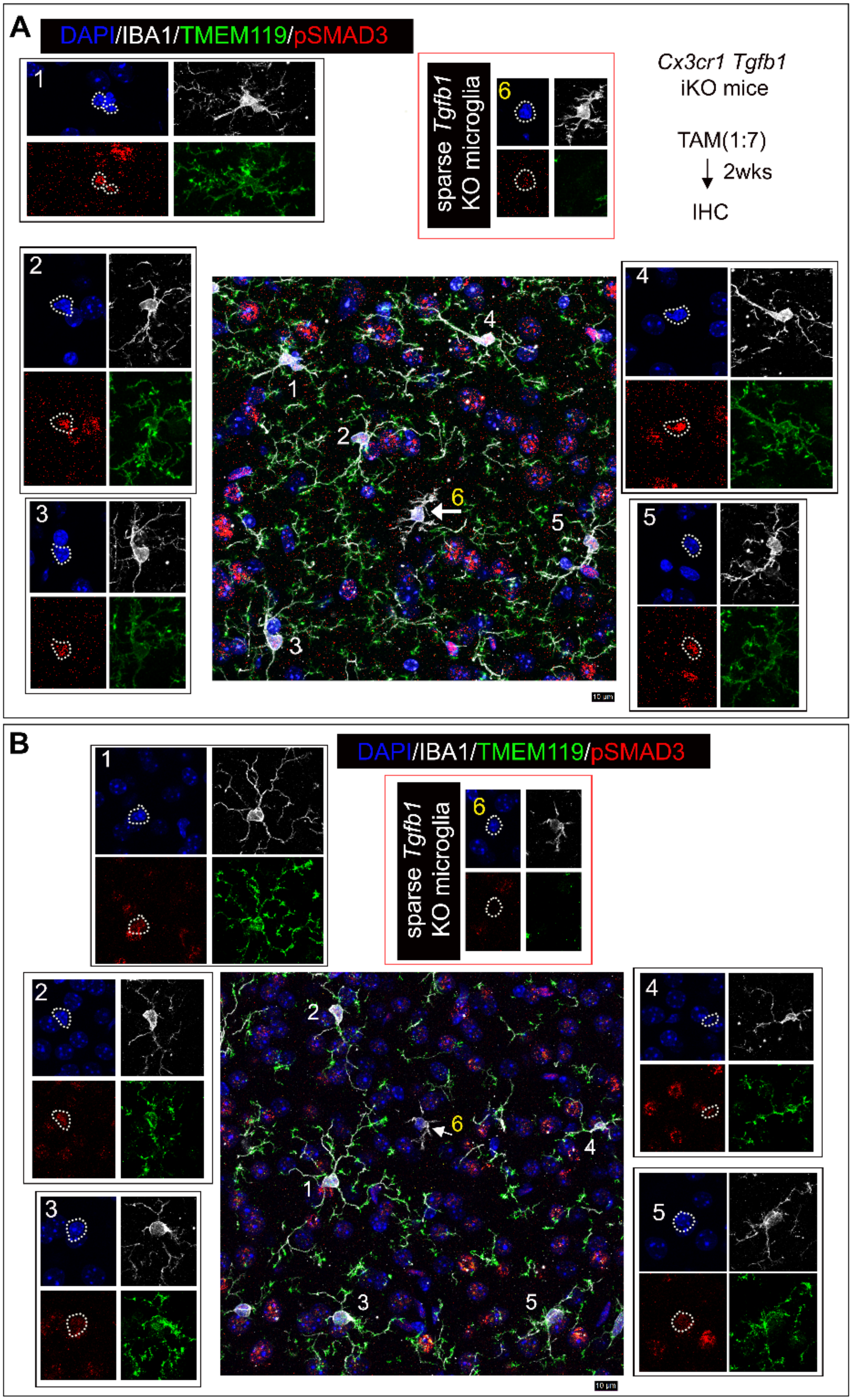
A
DAPI/IBA1/TMEM119/pSMAD3
Cx3cr1 Tgfb1
iKO mice
TAM(1:7)
2wks
IHC
sparse Tgfb1
KO microglia
1
2
3
4
5
6
10 μm
B
DAPI/IBA1/TMEM119/pSMAD3
sparse Tgfb1
KO microglia
1
2
3
4
5
6
10 μm

Supplemental Figure 12. Additional representative pSMAD3 immunostaining labeling confirms the downregulation of TGF-β downstream signaling in dyshomeostatic individual microglia in the sparse *Tgfb1* iKO model. (A) Representative image showing co-immunohistochemical staining with DAPI, IBA1, TMEM119, and pSMAD3. (A1-5) Surrounding normal microglia showing TMEM119 expression and pSMAD3 immunostaining. (A6) a single microglia cell with loss of TMEM119 expression and loss of pSMAD3 labeling. White arrow (microglia #6) marks the sparse recombined individual iKO microglia. (B) Representative image showing co-immunohistochemical staining with DAPI, IBA1, TMEM119, and pSMAD3. (B1-5) Surrounding normal microglia showing TMEM119 expression and pSMAD3 immunostaining. (B6) a single microglia cell with loss of TMEM119 expression and loss of pSMAD3 labeling. White arrow (microglia #6) marks the sparse recombined individual iKO microglia. Quantifications are presented in Fig 6. Representative results from n=6 iKO mice. Scale bar = 10μm.

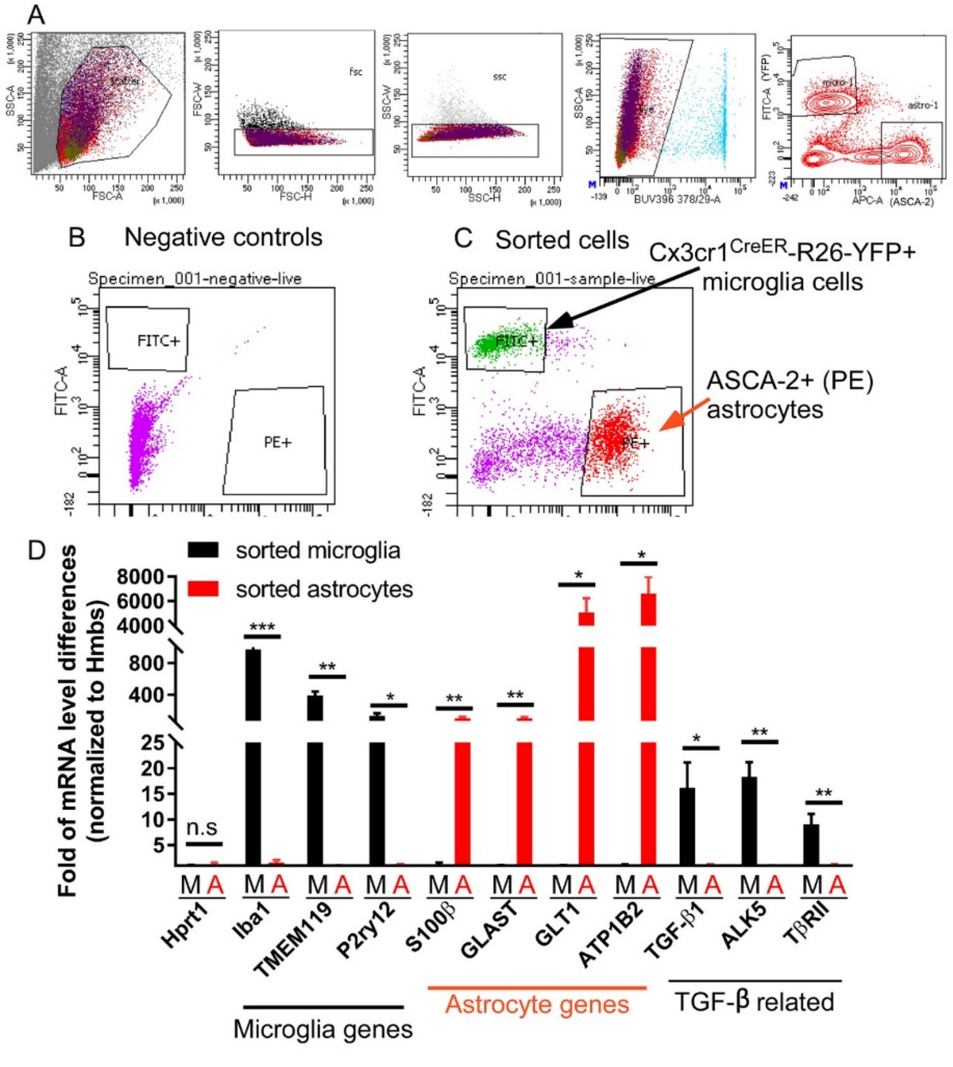

Supplementary Figure 13. Simultaneous sorting of microglia and astrocyte from the same mouse brain and subsequent RNA extraction and qRT-PCR supporting efficient sorting of microglia and astrocytes from the same brain. (A) Gating strategies for isolation of microglia based on TAM-induced R26-YFP expression and ASCA-2 staining on astrocytes. (B) Examples of unstained samples from a non-TAM treated and non-immunostained brain sample (left) and (C) TAM-treated *Cx3cr1^{CreER}-R26-YFP* mice that is stained with ASCA-2 antibodies. (D) The purity of sorted cells was validated using qRT-PCR by cell type-specific marker expression for microglia and astrocytes respectively. Note that YFP+ cells will contain both microglia and BAMs. Also note that *Tgfb1* (ligand) and both type I (*Alk5*) and type II (*Tgfbr2*) receptors are substantially enriched in microglia compared to astrocytes. Mean+SE. *, **, and *** p< 0.05, 0.01, 0.001. Student's t-test 2-sided, n=4 brains.

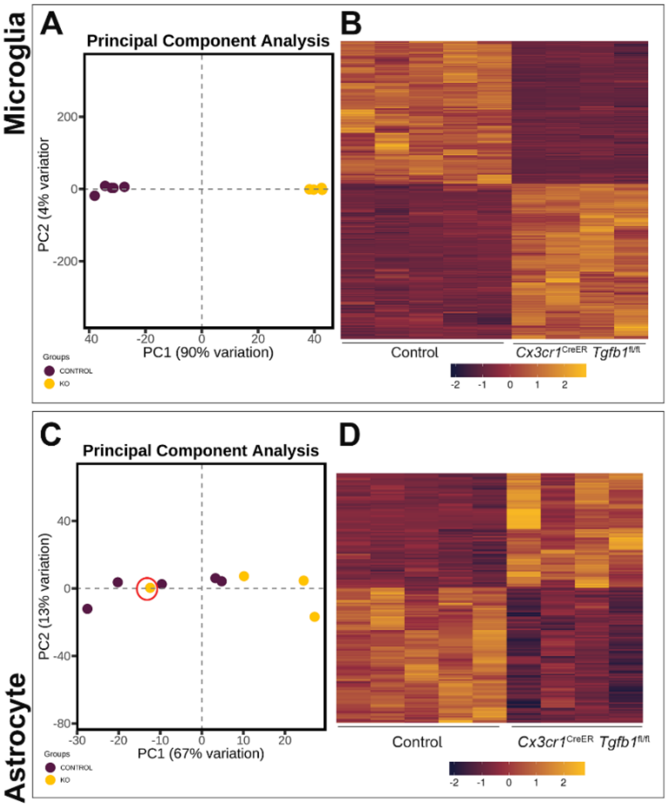

Supplemental Figure 14. Quality control comparisons using PCA analysis, heat maps of genetic changes. (A, C) PCA analysis plot of microglia and astrocyte samples. Note that one astrocyte sample from iKO mice clustered irregularly in the PCA plot which has an RNA Integrity Number (RIN) below 8 (red circle). (B, D) Heatmap of expression of significantly differentially expressed genes in microglia and astrocytes from control and iKO samples.

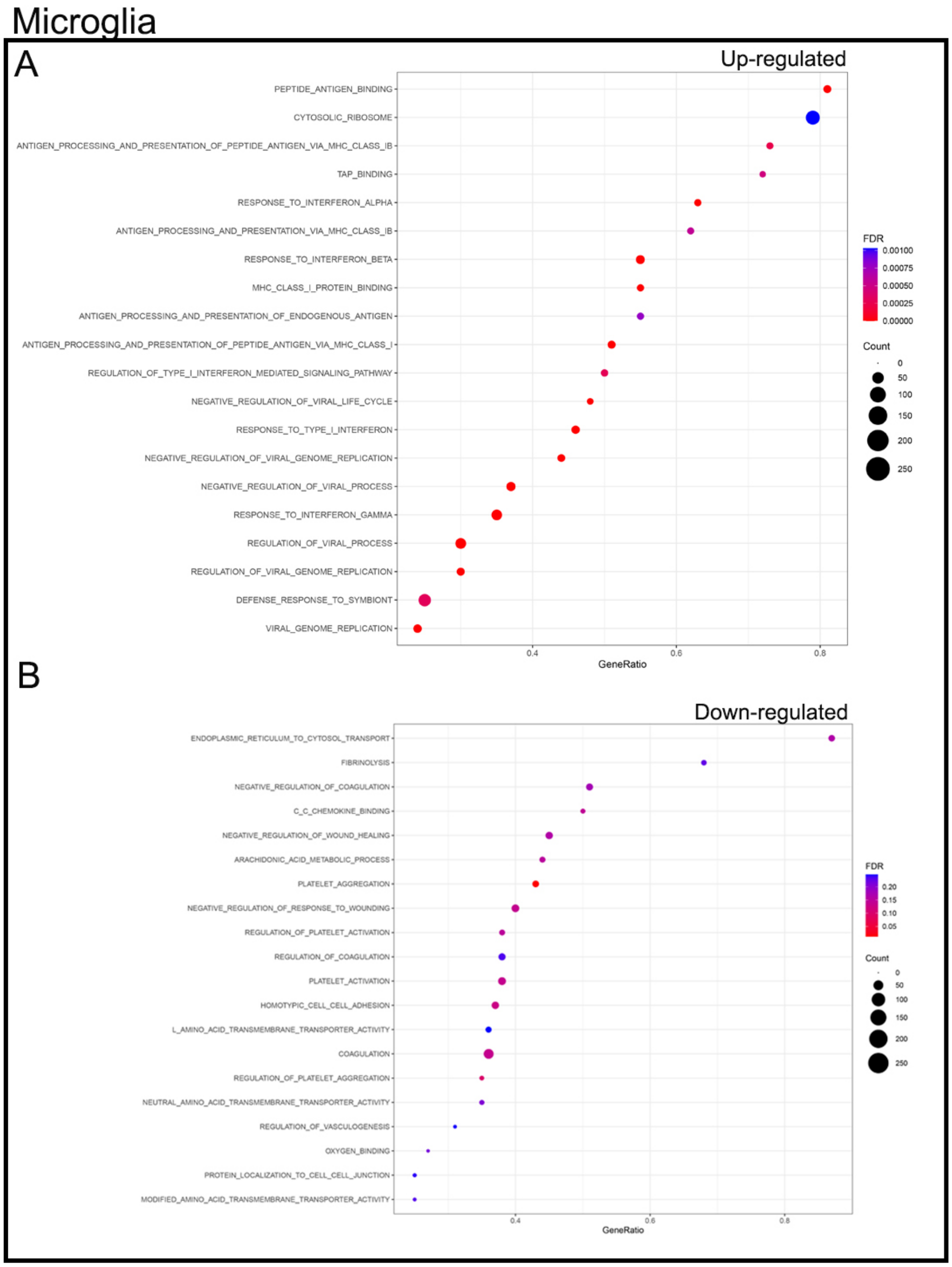

Supplementary Figure 15. GSEA Analysis of bulk RNA-sequencing data set examining GO pathways in microglia from *Cx3cr1*$^{\mathrm{CreER(Jung)}}$*Tgfb1*$^{\mathrm{fl/fl}}$ iKO mice compared to controls. (A) Up-regulated and (B) down-regualted GO pathways in brain microglia three weeks after loss of microglial *Tgfb1*.

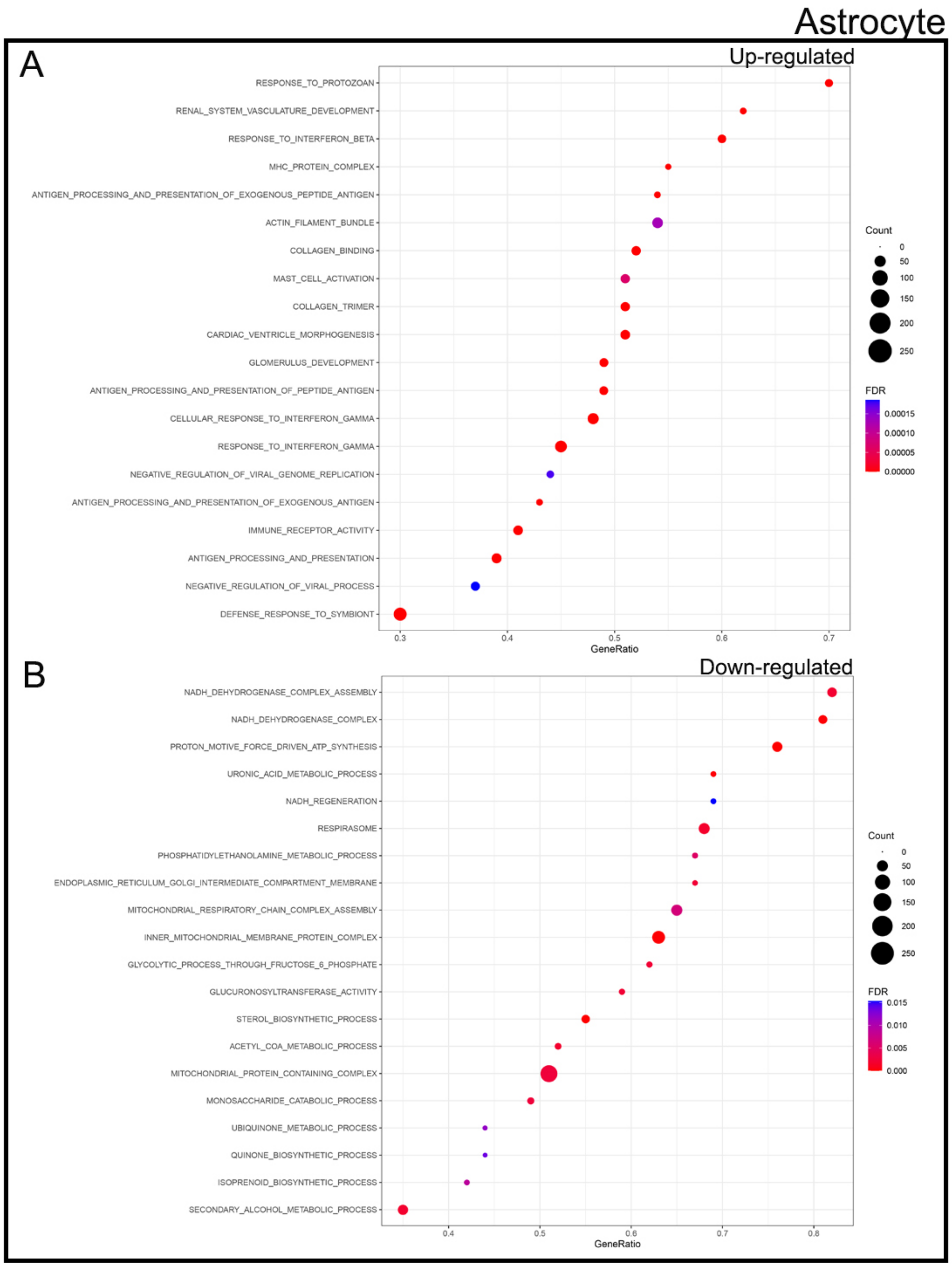

Supplementary Figure 16. GSEA Analysis of bulk RNA-sequencing data set examining GO pathways in astrocytes from *Cx3cr1*$^{\text{CreER(Jung)}}$*Tgfb1*$^{\text{fl/fl}}$ compared to controls. (A) Up-regulated and (B) down-regulated GO pathways of astrocytes from MG-*Tgfb1* iKO mice.

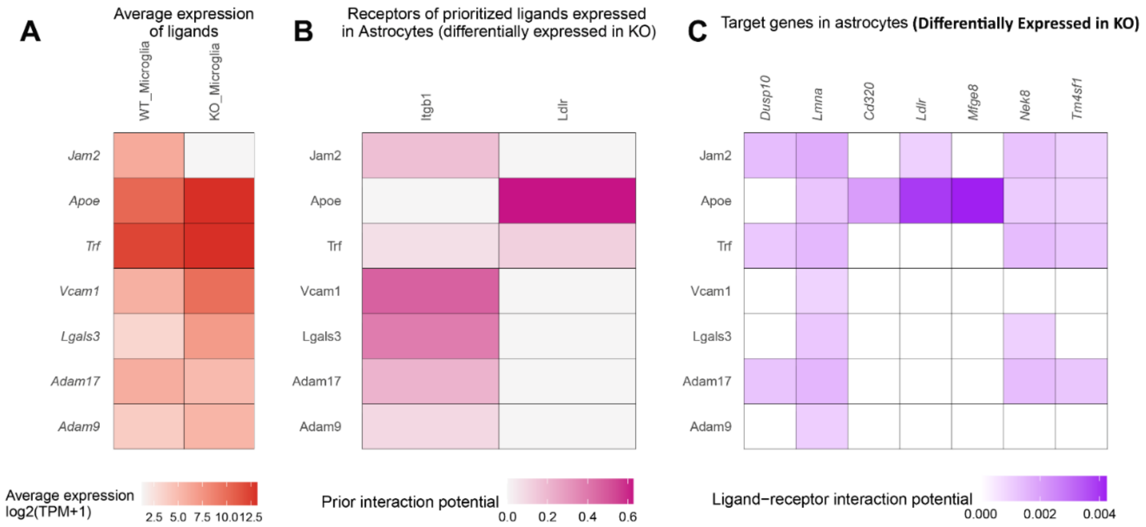

Supplementary Figure 17. NicheNet analysis to predict microglia ligand-astrocytes receptor-astrocyte target genes of the ligand-receptor mediated signaling in the *Cx3cr1^{CreER}Tgfb1* iKO mice at 3 weeks post TAM treatment. The NicheNet curated signaling pathways with their respective components were used to analyze the RNA-seq data and show (A) differentially expressed ligand genes from microglia, (B) the potential for interaction between the differentially expressed microglia ligands with the differentially expressed astrocyte receptors, and (C) the potential downstream targets based on differentially expressed astrocyte genes for each differentially expressed microglial ligand.

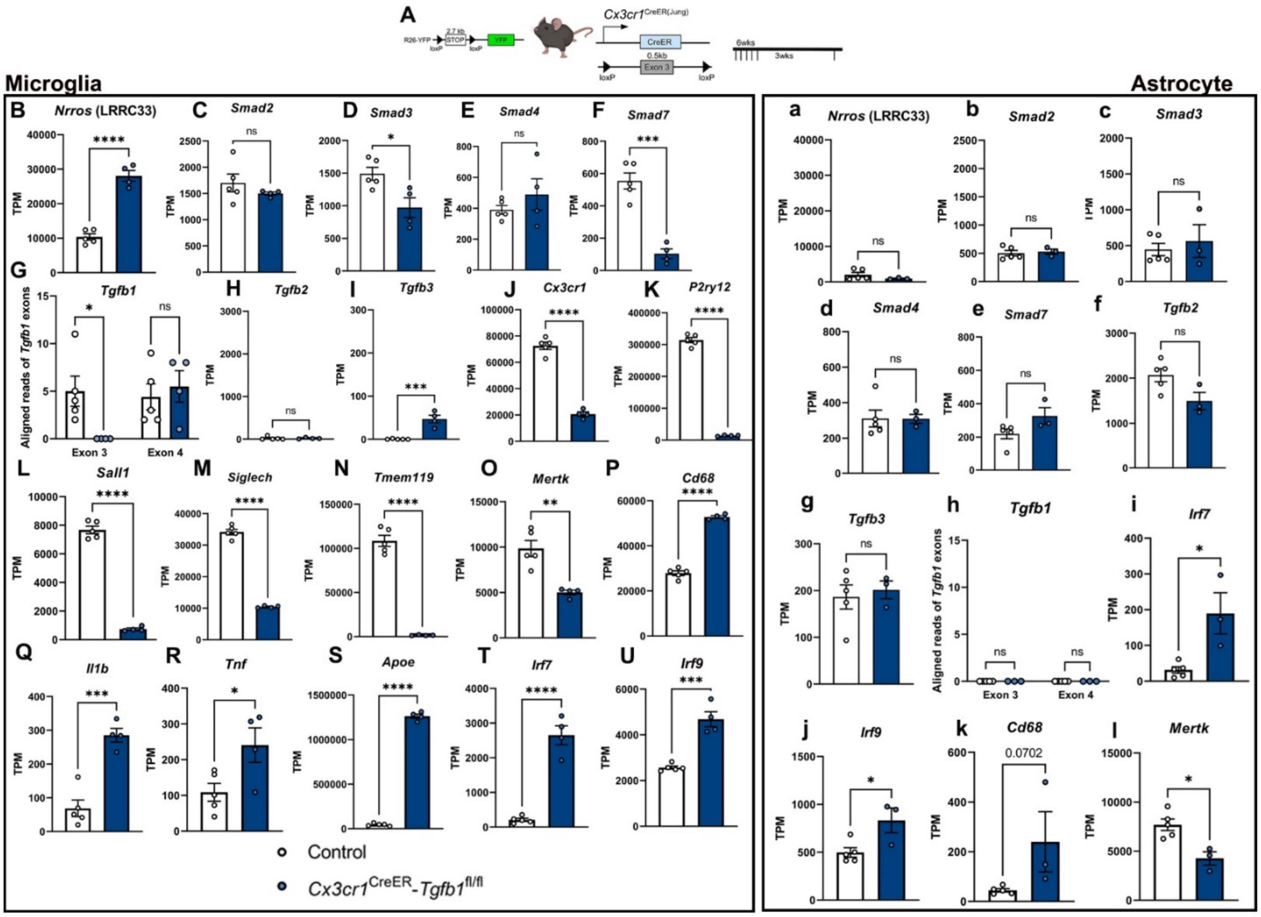

Supplementary Fig 18. **Gene expression changes observed in TGF-β signaling pathway component genes, microglia signature genes, and pro-inflammatory genes.** (A) Mouse model used. (B-I) Expression levels of TGF-β signaling components in control microglia and MG-*Tgfb1* iKO microglia. (J-O) Expression levels of microglia homeostatic signature genes and (P-U) pro-inflammatory genes. (a-h) Astrocytic expression of TGF-β signaling pathway components in control and MG-*Tgfb1* iKO mice. (i-l) Differentially expressed pro-inflammatory genes in astrocytes. Mean+SE. *, **, ***, **** p< 0.05, 0.01, 0.001, 0.0001. unpaired Student's t-test 2-sided, each data point represents a single animal. Supplementary Figure 18A was created with Biorender.com released under a Creative Commons Attribution-NonCommercial-NoDerivs 4.0 International license.

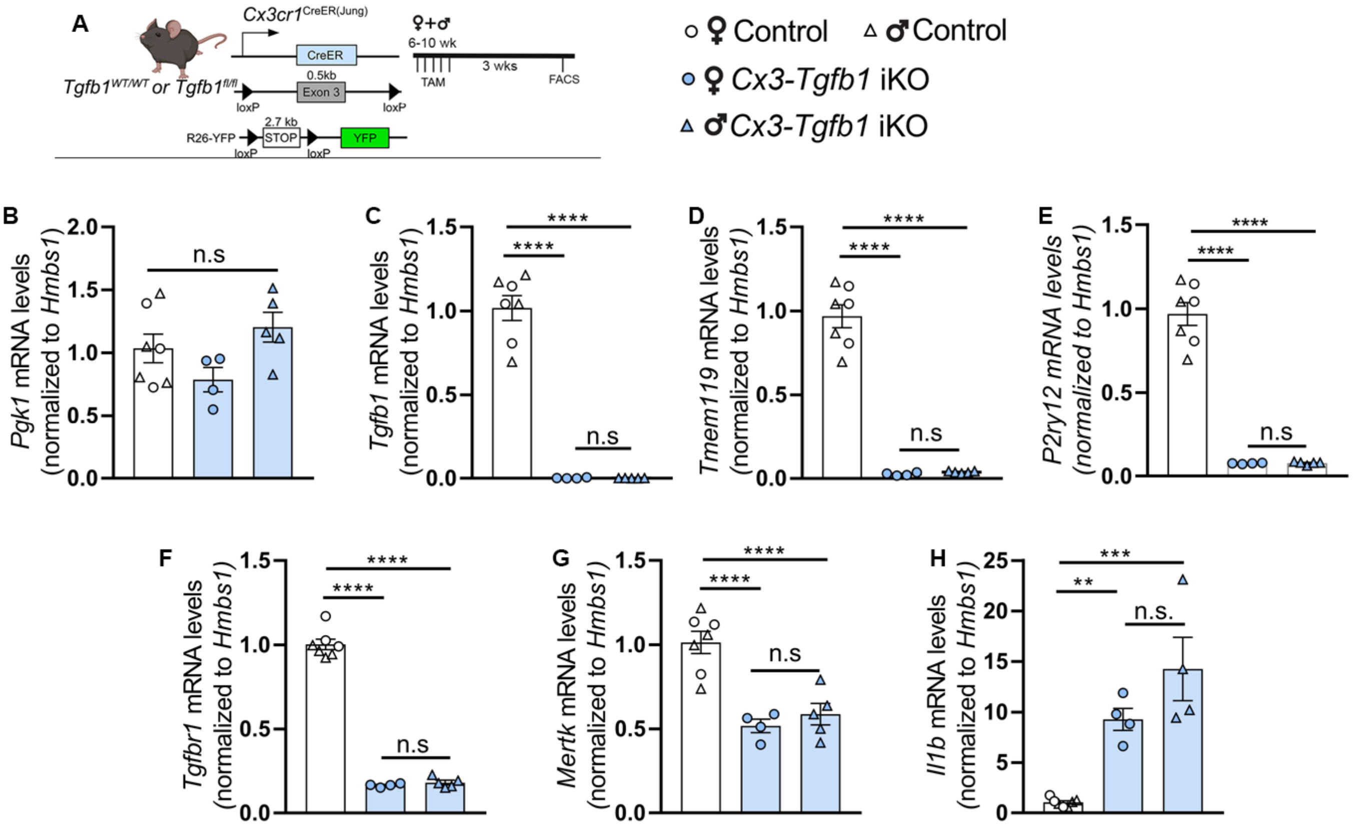

Supplementary Fig 19. **qRT-PCR from an independent cohort of female and male control and MG-*Tgfb1* iKO mice confirms similar gene dysregulation in both female and male iKO microglia**. (A) The mouse model used and the experimental timeline. FACS-isolated microglia were used for qRT-PCR analysis. (B-H) Expression levels of two house keeping genes (*Hmbs1* and *Pgk1*) remained the same in all groups while female and male knockout showed same upregulated or downregulated DEGs compared to control mice. Mean+SE. **, ***, **** p< 0.01, 0.001, 0.0001. One-way ANOVA 2-sided, each data point represents a single animal. Supplementary Figure 19A was created with Biorender.com released under a Creative Commons Attribution-NonCommercial-NoDerivs 4.0 International license.

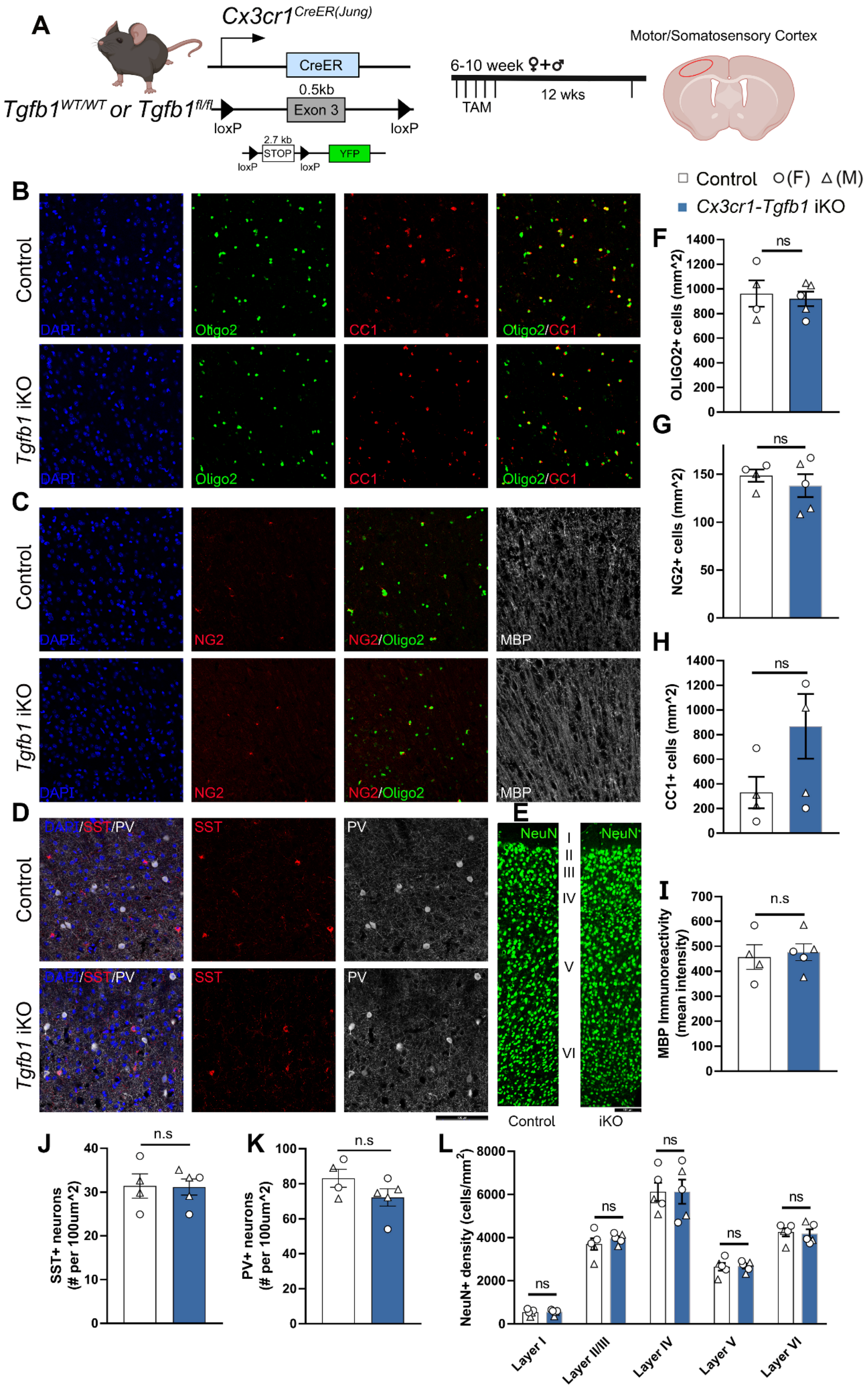
A
Cx3cr1 CreER(Jung)
CreER
0.5kb
Tgfb1 WT/WT or Tgfb1 fl/fl
Exon 3
loxP
loxP
2.7 kb
STOP
YFP
loxP
loxP
6-10 week ♀+♂
TAM
12 wks
Motor/Somatosensory Cortex
B
Control
Tgfb1 iKO
DAPI
Oligo2
CC1
Oligo2/CC1
C
NG2
NG2/Oligo2
MBP
D
DAPI/SST/PV
SST
PV
E
NeuN
I
II
III
IV
V
VI
Control
iKO
Control ○(F) △(M)
Cx3cr1-Tgfb1 iKO
F
OLIGO2+ cells (mm^2)
ns
G
NG2+ cells (mm^2)
ns
H
CC1+ cells (mm^2)
ns
I
MBP Immunoreactivity (mean intensity)
n.s
J
SST+ neurons (# per 100um^2)
n.s
K
PV+ neurons (# per 100um^2)
n.s
L
NeuN+ density (cells/mm^2)
ns
Layer I
Layer II/III
Layer IV
Layer V
Layer VI

Supplementary Figure 20. Microglia-specific *Tgfb1* gene deletion in the *Cx3cr1*$^{CreER(Jung)}$ driver does not affect myelination, oligodendrocyte lineage, NeuN+ neuronal or inhibitory interneuron populations in the cortical layers of the adult mouse brain at 12 weeks after TAM administration. (A) MG- iKO mouse model used and experimental timeline. (B, C, D and E) Representative immunohistochemistry images of OLIGO2, CC1, NG2, MBP, SST, PV, and NeuN in the cortex of control animals and Cx*3cr1*$^{CreER(Jung)}$*Tgfb1*$^{fl/fl}$ knockouts 12 weeks after tamoxifen administration. Quantification of different markers shown in (F-L). Mean+SE, ns= not significant. Unpaired Student's t-test 2-sided. Scale bar = 100μm. Supplementary Figure 20A was created with Biorender.com released under a Creative Commons Attribution-NonCommercial-NoDerivs 4.0 International license.

**Table S1 - Resources Table**		
REAGENT or RESOURCE	SOURCE	IDENTIFIER
Antibodies		
Goat Iba1 antibody	Abcam	Cat# ab5076, RRID: AB_2224402
Rabbit Iba1 antibody	Wako	Cat# 019-19741, RRID:AB_839504
Rabbit GFP antibody	Invitrogen	Cat# A11122, RRID: AB_268094
Chicken GFP antibody	Aves	Cat# GFP-1010, RRID: AB_2307313
Rat P2RY12 antibody	BioLegend	Cat#848002, RRID: AB_2650634
Rabbit anti-Mouse P2RY12 antibody	AnaSpec	Cat#55043A, RRID: AB_2298886
Rabbit anti-Green Fluorescent Protein (GFP) Polyclonal Antibody	Invitrogen	Cat#A11122, RRID: AB_221569
Recombinant Anti-Smad3 (phospho S423 + S425) antibody	Abcam	Cat# ab52903, RRID: AB_882596
GFAP	Sigma Aldrich	Cat# G3893, RRID: AB_477010
Rat MBP	BioRad	Cat# MCA409S, RRID: AB_325004
Rabbit NG2	Millipore	Cat# AB5320, RRID: AB_11213678
Goat olig2	RND Systems	Cat# AF2418, RRID:AB_2157554
Mouse CC1	Millipore Sigma	Cat# OP80, RRID: AB_2057371
Rabbit Parvalbumin	Swant	Cat# PV27, RRID:AB_2631173
Mouse Somatostatin	Santa Cruz	Cat# sc-55565, RRID:AB_831726
CD68	BioRad	Cat# MCA1957T, RRID: AB_2074849
NEUN	BioLegend	Cat# 834501, RRID: AB_2796508
Tgfb1 FACS antibody (clone TW7-16B4)	Biolegend	Cat# 141404, RRID:AB_10943504
Anti-mouse CD16/32 antibody	ThermoFisher	Cat#14-0161-86, RRID:AB_467135
CD45 antibody (clone 30-F11)	Biolegend	Cat# 103108, RRID:AB_312973
CD11b antibody (clone M1/70)	Biolegend	Cat# 101236, RRID:AB_312784
ASCA2 antibody, anti-mouse, APC	Miltenyi Biotec	Cat# 130-123-284, RRID: AB_2811488
Donkey anti-Goat IgG (H+L) Highly Cross-Adsorbed Secondary Antibody, Alexa Fluor™ Plus 488	Thermo Fisher Scientific	Cat# A32814, RRID: AB_2762838
Donkey anti-Goat IgG (H+L) Cross-Adsorbed Secondary Antibody, Alexa Fluor™ 555	Thermo Fisher Scientific	Cat# A-21432, RRID: AB_2535853

Cy3-AffiniPure Donkey Anti-Rat IgG (H+L)	Jackson ImmunoResearch	Cat# 712-165-153, RRID: AB_234066
Alexa Fluor 647-AffiniPure Donkey Anti-Rat IgG (H+L)	Jackson ImmunoResearch	Cat# 712-605-153, RRID: AB_2340694
Cy3-AffiniPure Donkey Anti-Rabbit IgG (H+L)	Jackson ImmunoResearch	Cat#715-165-152
Alexa Fluor 647-AffiniPure Donkey Anti-Rabbit IgG (H+L)	Jackson ImmunoResearch	Cat# 711-605-152, RRID: AB_2492288
Chemicals, peptides, and recombinant proteins		
Tamoxifen	Sigma	T5648-5G
Sunflower seed oil	Sigma	S5007
100% Ethanol	Sigma	493546-1L
Percoll	GE	17-0891-01
10x HBSS	Thermo Fisher Scientific	14185052
1x HBSS	Thermo Fisher Scientific	14175079
DAPI	Sigma	D9542
DNaseI	Roche	10104159001
Bovine Serum Albumin	Sigma	9048-46-8
Normal Donkey Serum	Jackson ImmunoResearch	017-000-121
Triton-100x	Sigma	T8787
2,2,2-Tribromoethanol	Sigma	T48402
2-Methyl-2-butanol	Sigma	240486
Deposited data		
RNA-seq data	This paper	GEO: GSE236032
RNA-seq data	Abud et al. 2017	GEO: GSE89189
Experimental Models: Organisms/strains		
Mouse: B6.129P2(Cg)-Cx3cr1tm2.1(cre/ERT2)Litt/WganJ	The Jackson Laboratory	IMSR Cat# 021160 RRID: IMSR_JAX:021160
Mouse: B6.129P2(C)-Cx3cr1tm2.1(cre/ERT2)Jung/J	The Jackson Laboratory	IMSR Cat# 020940 RRID: IMSR_JAX:020940
Mouse: B6(129S6)-P2ry12em1(icre/ERT2)Tda/J	The Jackson Laboratory	IMSR Cat# 034727 RRID: IMSR_JAX:034727
Mouse: C57BL/6-Tmem119em1(cre/ERT2)Gfng/J	The Jackson Laboratory	IMSR Cat# 031820 RRID: IMSR_JAX:031820
Mouse: B6.129X1-Gt(ROSA)26Sortm1(EYFP)Cos/J	The Jackson Laboratory	IMSR Cat# 006148 RRID: IMSR_JAX:006148
Mouse: C57BL/6J-*Tgfb1*em2Lutzy/Mmjax	The Jackson Laboratory	IMSR Cat# 065809-JAX RRID: MMRRC_065809-JAX

Oligonucleotieds		
Primers: Hbms (Probe #79)	Integrated DNA Technologies	F: TCCCTGAAGGATGTGCCTAC
Primers: Hbms (Probe #79)	Integrated DNA Technologies	R: ACA AGGGTTTTCCCGTTTG
Primers: Hprt1 (Probe #22)	Integrated DNA Technologies	F: TGATAGATCCATTCCTAT GACTGTAGA
Primers: Hprt1 (Probe #22)	Integrated DNA Technologies	R: AAGACATTCTTTCCAGTT AAA GTT GAG
Primers: Iba1 (Probe #3)	Integrated DNA Technologies	F: GGATTTGCAGGGAGGAAA A
Primers: Iba1 (Probe #3)	Integrated DNA Technologies	R: TGGGATCATCGAGGAATTG
Primers: Tmem119 (Probe #63)	Integrated DNA Technologies	F: AGGGAGCAAAGCCTGTGAA
Primers: Tmem119 (Probe #63)	Integrated DNA Technologies	R: TGACCGCTGAGCACCTCT
Primers: P2ry12 (Probe #102)	Integrated DNA Technologies	F: CCCGGAGACACT CATATCCTT
Primers: P2ry12 (Probe #102)	Integrated DNA Technologies	R: GTCCCAGGGGAGAAGGTG
Primers: S100β (Probe #78)	Integrated DNA Technologies	F: GACTCCAGCAGCAAAGGTG
Primers: S100β (Probe #78)	Integrated DNA Technologies	R: AGACATCAATGAGGGCA ACC
Primers: Glast (Probe #77)	Integrated DNA Technologies	F: CATTGCAGTGGACTGGTTTC
Primers: Glast (Probe #77)	Integrated DNA Technologies	R: CTGCTCCGAGGGAGTCAC
Primers: Glt1 (Probe #103)	Integrated DNA Technologies	F: GATGCCTTCCTGGATCTCATT
Primers: Glt1 (Probe #103)	Integrated DNA Technologies	R: TTGTCACTGTCTGAATCTG CTG
Primers: Atp1b2 (Probe#58)	Integrated DNA Technologies	F: AGCTCAACAAGTTCTTGGA ACC
Primers: Atp1b2 (Probe#58)	Integrated DNA Technologies	R: CTGGACGGCAGACATCATT
Critical commercial assays		
Papain dissociation kit	Worthington	Cat#LK003150
LIVE/DEAD fixable dead cell stain kit	Invitrogen	Cat# L34960
RNAqueous™-Micro Total RNA Isolation Kit	ThermoFisher	Cat#AM1931
iScript cDNA Synthesis Kit	BioRad	Cat#1708890
TaqMan fast advanced master mix	ThermoFisher	Cat#4444556

Tgfbr1 qrtPCR assay (Mm00436965_m1)	ThermoFisher	Cat#4351372
Tgfb1 qrtPCR assay (Mm03024053_m1)	ThermoFisher	Cat#4331182
Roche Universal Probe Library #79	Roche	Cat# UPL71THRU80
Roche Universal Probe Library #108	Roche	Cat# UPL101THRU110
Roche Universal Probe Library #3	Roche	Cat# UPL1THRU10
Software and Algorithms		
ImageJ	https://imagej.nih.gov/ij/	https://doi.org/10.1038/nmeth.2089 RRID:SCR_003070
Nikon Element	Nikon	V3.22, RRID: SCR_014329
Stereo Investigator Image Software	MBF Bioscience	V2022.2.1, RRID:SCR_002526
FlowJo X software	SciCrunch Registry	vX10, RRID:SCR_008520
Trim Galore!	Babraham Bioinformatics	v0.4.2, RRID:SCR_011847
Cutadapt	Martin, 2011	v1.9.1, RRID:SCR_011841
STAR	Dobin et al, 2012	RRID:SCR_004463
Sambamba	Tarasov et al. 2015	v0.6.8, https://doi.org/10.1093/bioinformatics/btv098
Subread	Liao et al. 2019	v1.6.2, RRID:SCR_009803
DESeq2	Love et al. 2014	v1.26.0, RRID:SCR_015687
Ggplot2	Wickham 2016	RRID:SCR_014601
GSEA	Subramanian et al. 2015	v3.0, RRID:SCR_003199
Biorender.com	Biorender	RRID:SCR_018361
Other		
Microscope	Leica	DM5000B
Confocal Microscope	Leica	Stellaris 8
FACS	BD Biosciences	BD/FACSAria II
Flow Cytometry	BD Biosciences	BD Canto II
