# Supplementary material for: Adult microglial TGFβ1 is required for microglia homeostasis via an autocrine mechanism to maintain cognitive function in mice
Source: Nat Commun. 2024 Jun 21;15:5306. doi: 10.1038/s41467-024-49596-0 (PMC11192737; doi:10.1038/s41467-024-49596-0)
Supplement: Supplementary file 2 — Description of Additional Supplementary Files [file 41467_2024_49596_MOESM2_ESM.docx]

**Description of Additional Supplementary Files**

**Supplementary Data 1:** Differentially expressed genes from sorted microglia collected using YFP reporter via FACS from Cx3cr1CreER(Jung)Tgfb1wt/wt and Cx3cr1CreER(Jung)Tgfb1fl/fl. Related to Fig 7.

**Supplementary Data 2:** Differentially expressed genes from sorted astrocytes collected using ASCA-2 antibody labeling via FACS from Cx3cr1CreER(Jung)Tgfb1wt/wt and Cx3cr1CreER(Jung)Tgfb1fl/fl. Related to Fig 7.

**Supplementary Data 3:** GSEA showing negatively regulated pathways from microglia from the RNA-seq data collected using Cx3cr1CreER(Jung)Tgfb1wt/wt and Cx3cr1CreER(Jung)Tgfb1fl/fl. Related to Fig 7 and supplementary Fig 15.

**Supplementary Data 4:** GSEA showing positively regulated pathways from microglia from the RNA-seq data collected using Cx3cr1CreER(Jung)Tgfb1wt/wt and Cx3cr1CreER(Jung)Tgfb1fl/fl. Related to Fig 7 and supplementary Fig 15.

**Supplementary Data 5:** GSEA showing negatively regulated pathways from astrocytes from the RNA-seq data collected using Cx3cr1CreER(Jung)Tgfb1wt/wt and Cx3cr1CreER(Jung)Tgfb1fl/fl. Related to Fig 7 and supplementary Fig 16.

**Supplementary Data 6:** GSEA showing positively regulated pathways from astrocytes from the RNA-seq data collected using Cx3cr1CreER(Jung)Tgfb1wt/wt and Cx3cr1CreER(Jung)Tgfb1fl/fl. Related to Fig 7 and supplementary Fig 16.

**Supplementary Data 7:** GO biological processes downregulated in the absence of TGF-β ligand present in the RNA-seq data collected from Cx3cr1CreER(Jung)Tgfb1wt/wt and Cx3cr1CreER(Jung)Tgfb1fl/fl mice and human microglia like cells derived from iPSCs underwent 24 hours of TGF-β ligand withdrawal. GO analysis was performed using the Enrichr online database. P values were calculated using Fisher’s exact test, and adjustments for multiple comparisons were made using the Benjamini-Hochberg method.

**Supplementary Video 1**. 3D rendered confocal image stack of coimmunostaining of IBA1 (red) and TMEM119 (white) in sparse MG-Tgfb1 iKO brain showing a single morphologically altered and IBA1+/TMEM119- KO

microglia surrounded by normal neighboring microglia (IBA1+/TMEM119+).
